# Engineering principles of zwitterionic hydrogels: Molecular architecture to manufacturing innovations for advanced healthcare materials

**DOI:** 10.1016/j.mtbio.2025.102085

**Published:** 2025-07-12

**Authors:** Hoang Linh Bui, Hoang Nam Nguyen, Jui-Yang Lai, Chun-Jen Huang

**Affiliations:** a Faculty of Biotechnology, Chemistry, and Environmental Engineering, PHENIKAA School of Engineering, PHENIKAA University, Hanoi, 12116, Viet Nam; b Faculty of Automation Engineering, Can Tho University, 3/2 Street, Can Tho city, 900000, Viet Nam; c Department of Biomedical Engineering, Chang Gung University, Taoyuan City, 33302, Taiwan, ROC; d Department of Ophthalmology, Chang Gung Memorial Hospital, Linkou, Taoyuan City, 33305, Taiwan, ROC; e Department of Materials Engineering, Ming Chi University of Technology, New Taipei City, 24301, Taiwan, ROC; f Center for Drug Research and Development, College of Human Ecology, Chang Gung University of Science and Technology, Taoyuan City, 33303, Taiwan, ROC; g Center for Biomedical Engineering, Chang Gung University, Taoyuan City, 33302, Taiwan, ROC; h Department of Chemical and Materials Engineering, National Central University, Chungli County, Taoyuan City, 32049, Taiwan, ROC; i R&D Center for Membrane Technology, Chung Yuan Christian University, 200 Chung Pei Rd., Chungli County, Taoyuan City, 32023, Taiwan, ROC; j School of Materials Science and Engineering, The University of New South Wales, Sydney, NSW, 2052, Australia

**Keywords:** Hydrogel, Biomedical applications, Molecular design, Preparation process, Zwitterionic

## Abstract

Zwitterionic hydrogels have garnered significant attention for their exceptional anti-fouling properties and biocompatibility. These materials have been further enhanced through the incorporation of highly crosslinked 3D networks, endowing them with novel physicochemical characteristics such as stimuli-responsive swelling, ion conductivity, and antibacterial capabilities. In certain cases, zwitterionic motifs can impart self-healing properties for hydrogels, potentially paving the way for the development of functional biomaterials that closely mimic biological tissues. In recent years, we have witnessed substantial efforts from the scientific community to harness the potential of zwitterionic hydrogels for various biomedical applications. This review offers a comprehensive examination of the design principles underlying these materials, spanning from molecular engineering aspects to recent advancements in synthesis routes and fabrication methods. The discussion encompasses the advantages and limitations of different preparation processes, as well as prospects for zwitterionic hydrogels. As a result, this review aims to provide valuable insights for researchers and engineers working to optimize zwitterionic hydrogels for specific biomedical applications, including drug delivery, tissue engineering, and biosensors.

## Graphical abstract

**Figure:**
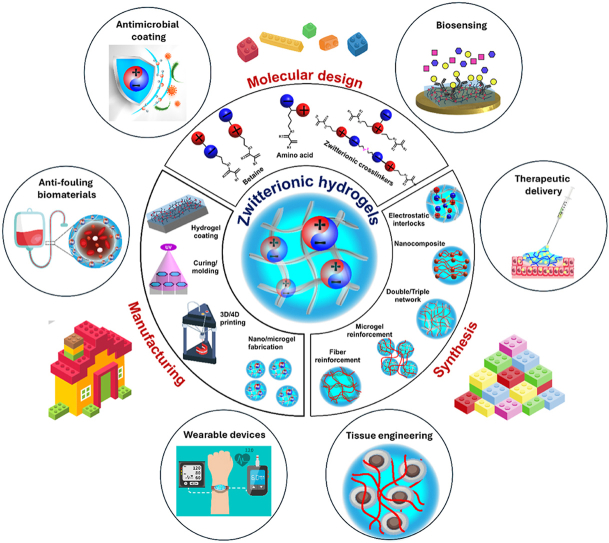

## 1 Introduction

Zwitterionic materials represent a unique class of ionic systems characterized by their charge neutrality, achieved through the incorporation of both anionic and cationic moieties within a single monomer unit. Accordingly, these materials have superior hydrophilicity, which is attributed to their capacity to form robust hydration shells via strong ionic solvation [1]. Moreover, their enhanced hydrophilicity was considered to surpass that of conventional non-ionic hydrophilic polymers such as polyethylene glycol, polyvinyl alcohol, and poly(2-methyl-2-oxazoline) [2]. As a result, the distinctive properties of zwitterionic materials confer several advantages in biomedical applications. The first benefit is their remarkable ability to mitigate nonspecific protein adsorption, a critical factor in preventing biofouling and maintaining the integrity of biomedical surfaces. Fouling resistance, coupled with high biocompatibility, negligible immunogenicity, chemical stability, and versatility, renders zwitterionic materials particularly suitable for the development of functional biomaterials [1,3,4]. Over the past few years, there has been a surge in interest in zwitterionic materials in biomedical applications. From large-scale medical devices to nano-scale drug delivery systems, zwitterionic agents have been integrated in various forms, including surface coatings, implants, biosensors, separation membranes, and nanotheranostic carriers [[5], [6], [7], [8]].

**Fig. 1:**
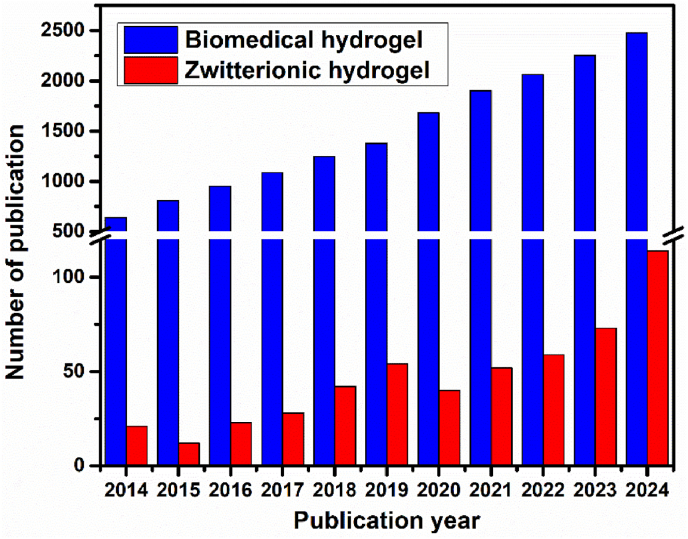
Annual numbers of publications searched by the key words “biomedical hydrogel” and “zwitterionic hydrogel” according to Pubmed database.

Zwitterionic hydrogels are a type of polymeric network composed of zwitterion residues. Their high-water content and porous structure give the hydrogels viscoelasticity similar to natural soft tissues, along with permeability to a wide range of chemical and biological molecules. Beyond physical resemblance, zwitterionic hydrogels also share chemical similarities with biological tissue, as cellular membranes contain zwitterionic motifs such as phosphatidylcholine in their phospholipid bilayers [9]. Consequently, considerable efforts have been directed toward investigating zwitterionic hydrogels as functional biomaterials [[10], [11], [12], [13]]. Accordingly, the zwitterionic hydrogels have depicted a growing interest in biomedical engineering, which is evident by the elevated publications in the past 10 years (Fig. 1) (according to Pubmed database).

Despite their numerous advantages, zwitterionic material systems still face inherent challenges primarily stemming from their tendency of excessive swelling, which often results in compromised mechanical properties. This limitation has become a focal point of research, with significant efforts directed towards developing viable solutions. Thus, there is a critical need for robust hydrogels capable of withstanding deformation, maintaining structural integrity, and resisting mechanical failure under physiological stresses for biomedical devices. The requirements are particularly pertinent for long-term implants and sustained drug delivery systems, where the hydrogel must retain its bulk and surface properties over extended periods. Further limitations in the development of zwitterionic hydrogel arise from synthesis and fabrication considerations. From an economic perspective, the price per gram of marketed zwitterionic monomers representing sulfobetaine (CAS number 3637-26-1**)** and phosphobetaine (CAS number 67881-98-5) motifs are approximately 1.6 and 64 USD according to the quotation in the Tokyo Chemical Inc., which are higher than the other hydrophilic counterpart, such as 2-hydroxyethyl methacrylate (HEMA) (CAS number 868-77-9**)** used for contact lenses (0.84 USD). However, the cost-effective relation between synthesis methods, fabrication techniques, and functionality of zwitterionic hydrogels has not been carefully considered. For these reasons, multiple reviews have been dedicated to introducing and classifying current engineering approaches for zwitterionic hydrogels [1,12,14]. Although zwitterionic hydrogels are often highlighted for their anti-fouling properties, the relationship between the selection of zwitterionic motifs and the practical considerations of fabrication techniques for functional biomaterials has not been extensively explored.

In this review, our primary scope is to deliver the current advances in zwitterionic hydrogels for biomedical applications and present a comprehensive summary of the recent design principles of zwitterionic hydrogels, primarily aiming to address the major challenges in their production process. We began by briefly introducing the zwitterionic motifs for hydrogel synthesis and discussing their relevance in the biomedical field. Additionally, we explore the insights these design strategies offer regarding their synthesis pathways and fabrication techniques, with a focus on their applicability in biomedical fields. Overall, we envision that the practical viewpoints provided in this review can help form a bridge that closes the gaps between lab-scale research and the wider recognition and adoption of zwitterionic hydrogels at an industrial scale.

## 2 Design rationale in zwitterion selection

In this section, the representative zwitterionic materials will be summarized and classified into three groups: group 1 consists of commonly known motifs in the betaine family; group 2 consists of amino acid-based zwitterionic motifs; group 3 consists of other derivatives. In each subsection, the unique features of these motifs will be summarized to highlight their relevance in biomedical applications.

### 2.1 Betaine motifs

In general, betaine motifs (phosphocholine (PC), sulfobetaine (SB) and carboxyl betaine (CB)) are characterized by their unique structural compositions, bestowing these polymers with several key properties, including hydration and anti-fouling performance. In this context, it is important to note that the extent to which these properties are exhibited can vary significantly based on the hydration levels of their negatively charged groups. Particularly, when comparing the hydration strength of CB and SB motifs with identical N(CH_3_)_3_^+^ groups, the key difference lies in their anionic groups: CB has two oxygen atoms in the carboxyl group while SB has three in the sulfonate group. This structural difference indicates that SB attracts more water molecules due to its larger coordination shell volume. However, oxygen atoms of CB carry higher partial charges than those of SB, resulting in stronger individual water interactions. Consequently, while SB binds more water overall, CB forms more stable water retention and preferential hydration dynamics [15]. Nevertheless, due to the highly swollen and stretched zwitterionic polymeric networks, bio-foulants are able to penetrate and stagnate even without interfacial attachment. Consequently, when selecting a betaine motif for anti-fouling applications, a broader range of factors beyond just hydration capability, including stimuli responsiveness and crosslinking capability, precursor composition, and preparation process, are often carefully considered. Please refer to the comprehensive reviews for more details on the influence of hydration dynamics on the anti-fouling performance of polybetaine [1,14,16].

**Fig. 2:**
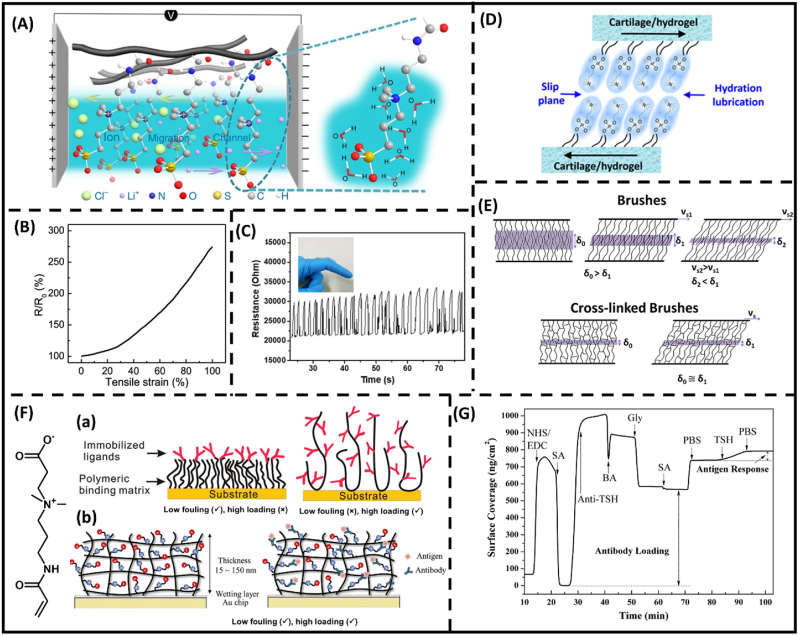
**Betaine motifs.** (A) Schematic illustration of SB-based gel applied on electrodes [45]. (B) Resistance ratio–strain curve of the SB-based hydrogel [24]. (C) Photographs of the SB hydrogel-based pressure sensor of continuous finger bending [25]. (D) Schematic demonstration of hydration lubrication of hydrogels at slip plane [32]. (E) Differences between polymer brush and crosslinked counterpart under variation of shear velocity [35]. (F) (a) The superiority of one-layer 2D CB-based hydrogels to the commercial dextran platform in terms of fouling resistance and ligand loading capability. (b) The optimization of hydrogel thickness and crosslinking density for enhanced anti-fouling properties and functionalization [40,42,44] (G) Typical surface plasmon resonance (SPR) sensorgram for anti-body immobilization and antigen detection on CB-based hydrogels [44].

Among all the betaine motifs, SB monomer, such as sulfobetaine methacrylate, is considered a favorable choice as a major component due to its easy accessibility. The two key features of poly(sulfobetaine) (PSB) hydrogels have recently been explored including their ionic conductivity and self-healing capability. Specifically, ionic conductivity is an important property for biomedical applications (bio-electronics, bio-actuators, and neuroscience) [[17], [18], [19]], in which zwitterions present as the charge carriers, maneuvering the mobility of ions and affecting the functionality of integrated devices depending on ionic strength of the solution as demonstrated in Fig. 2A. Compared with other betaine motifs, PSB hydrogels with higher polarity were suggested to promote fast ion dissociation and transportation upon increasing electrolyte concentration, resulting in better high ionic conductivity [20,21]. In addition, the dipole-dipole interactions between the SB moieties allow the PSB hydrogel network to rapidly reform and heal after being damaged or deformed through zwitterionic fusion [22,23]. In particular, the tensile strain of SB-based hydrogels exhibited linear relationship with electronic resistance as shown in Fig. 2B [24]. In combination with the ionic conductivity and mechanical flexibility, the signals recorded from the hydrogel can be interpreted through distinct patterns of electrical resistance (Fig. 2C) [25]. Note that, PSB polymers exhibit conformational changes in response to multiple stimuli (pH, salt, and temperature) [26]. The phenomena are attributed to the charge−charge or dipole−dipole interactions, consequently making SB motif and their derivatives extensively attractive to sensing applications.

The strong hydration of the phosphorylcholine (PC) headgroups in PMPC impedes and reorients adsorbed water molecules, providing stable anti-fouling properties against the change in ion strength as compared to that of SB [27,28]. Specifically, the anionic charge density and carbon spacer length between charged groups of PC are the main factors influencing the fouling-resistance capability [29,30]. In the hydrogel format, the friction force between the hydration interfaces and substrate surfaces is further dissipated through elastic deformation of polymer chains [31,32]. Furthermore, owing to the strong hydration layer, the highly-ordered water molecules (referring as “freezing water”) surrounding the PC heads in PMPC induced exceptional hydration repulsion, generating deficient friction force between hydrated slip planes (Fig. 2D) even under high shear force and high ionic conditions [[32], [33], [34]]. Notably, Klein and colleagues have highlighted the dynamic response of crosslinked PMPC to shear rate, in which hydrogel formation exhibited lower friction force at low shear velocity, which is more superior to PMPC brushes (Fig. 2E) [35]. Furthermore, shear force applied on hydrogels can be concentrated at crosslinking sites. Hence, lubricity of crosslinked conformation is attributed to the resilience through the additional energy dissipation at crosslinking sites.

The anti-fouling property of zwitterionic hydrogels can make them bioinert, underscoring the need for functionalization approaches like incorporating CB moieties to enhance their versatility for specific biomedical requirements. The carboxyl groups within CB exhibit a significantly higher pKa than phosphate and sulfate groups in PC and SB, respectively. Its protonation can be influenced by the surrounding chemical conditions, allowing covalent conjugation between carboxybetaine hydrogel and a variety of biomolecules, such as proteins, peptides, or therapeutic agents containing coupling moieties. Generally, bio-conjugation techniques of CB involve carbodiimide reactions. In these methods, the coupling between CB groups with biomacromolecules takes place by activating carboxylic acid residues under 1-ethyl-3-(3-dimethylaminopropyl)carbodiimide and N-hydroxysuccinimide (EDC/NHS) activation chemistry [[36], [37], [38], [39]]. The intermediate then reacts with amino groups present on biomolecules, establishing a durable connection between the hydrogel and the bioactive molecules as demonstrated in Fig. 2F. Bioconjugations of the CB motif provide specific surface binding sites, which are highly desirable in biosensor development [[40], [41], [42], [43], [44]]. In this implementation, CB hydrogel platforms exhibit multiple unique qualities to improve sensor sensitivity and functionalities (Fig. 2G), including resistance against nonspecific protein adsorption in undiluted whole blood [40], and improve high ligand loading [44].

### 2.2 Amino acid-based motifs

**Fig. 3:**
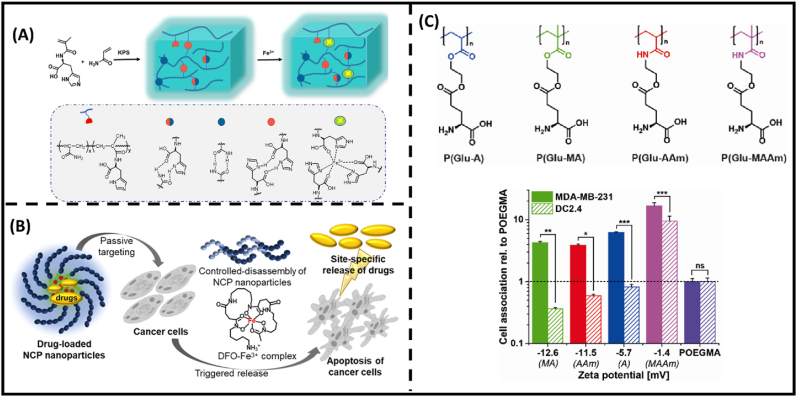
**Amino-acid motifs** (A) The network structure of HisMA hydrogel and the P(AM-HisMA)-Fe^3+^ hydrogel [46]. (B) Schematic illustration of curcumin-loaded SerA-based nanogels toward breast cancer treatment [52]. (C) Chemical structures of Glu-derived zwitterionic hydrogels and their cell specificity relative to POEGMA hydrogels as non-ionic control [55].

Natural amino acids represent the building blocks of proteins, which are necessary for the construction of proper cellular structure and support of cellular survival via biocatalytic transformations. Drawing inspiration from nature, tailoring biomolecule-derived materials that mirror the function and structure of natural proteins is significant in transforming hydrogel into soft tissue replacement. To this end, polymers obtaining tethered peptide bonds (amino and carboxyl residues) not only replicate bio-active functions found in living organisms but also can enhance the mechanical structure of hydrogels. This improvement is achieved by establishing dynamic interactions, such as hydrogen bonding, hydrophobic interaction, electrostatic interaction, π–π stacking, and dynamic covalent reactions, such as disulfide bonds, Schiff base reaction, and Michael addition. Accordingly, the properties confer an advantageous edge for amino acid-based polymeric materials compared with other zwitterions in biological mimicry. In particular, histidine (His), cysteine (Cys), serine (Ser) and lysine (Lys) have been used for their complexation capability with metal ions [[46], [47], [48], [49], [50]]. At low pH conditions, both carboxylate and amine groups of the amino acid are protonated, which leads to positive surface charge of the hydrogel [51]. At a wide pH range (from pH 5 to 9), both carboxylate and amine groups reach charge neutrality, facilitating anti-fouling capability. Furthermore, implementation of amino acid-derived gels is also realized by forming a coordination bond with multivalent metal ions such as Cu^2+^ and Fe^3+^, providing functionalities for mechano-sensing actuators and site specific drug release (Fig. 3A) [46,47]. For site-specific delivery, Ser-based hydrogels were found to coordinate with a wide spectrum of metal ions. This leads to the formation of spherical nanogel aggregates, caging cytotoxic effects of metal ions in circulating system and facilitating on-demand drug release in cancer pharmacotherapy (Fig. 3B) [48,49,52]. Similarly, a diblock polymer composed both Lys has shown effectiveness in copper detoxification [50]. The self-assembly of coordination complex can also allow improvement in drug payload and carrier stability [52]. The utilization of glutamic acid-inspired polyzwitterion as a conjugate in drug carriers is aimed at enhancing the effectiveness of anticancer drugs while reducing their toxicity towards normal cells [53,54]. Recently, the backbone configuration of the bioinspired polyzwitterion was evaluated for its cellular specificity [55]. A range of glutamate-derived monomers featuring distinct backbones, including acrylate, methacrylate (MA), acrylamide (AAm), and methacrylamide (MAAm) were examined [55]. This variation significantly influences the drug uptake of cancer cells (Fig. 3C).

### 2.3 Emerging zwitterionic molecules

**Fig. 4:**
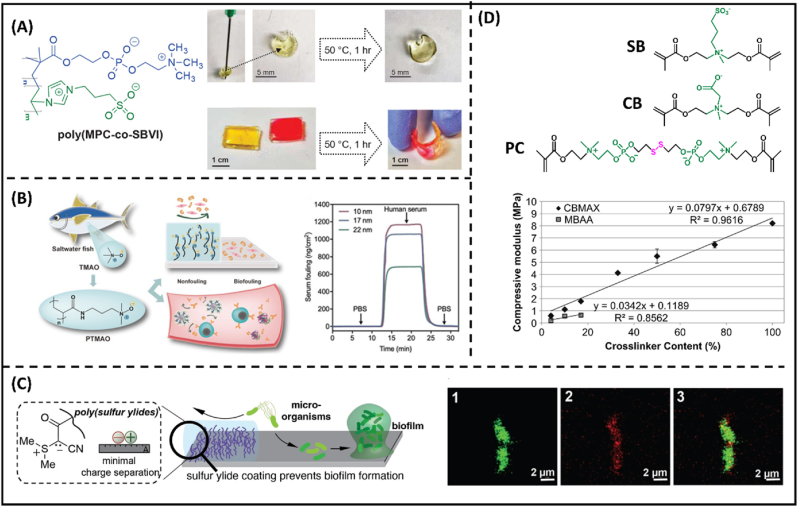
**Emerging zwitterionic molecules.** (A) Chemical formula of a fully zwitterionic hydrogels consisting of 2-methacryloyloxyethyl phosphorylcholine (MPC) monomer and sulfobetaine vinylimidazole (SBVI) monomer, and the digital images demonstrating their self-healing capability [64]. (B) The design of TMAO hydrogel derived from TMAO monomer and SPR sensorgram of TMAO films [58]. (C) Schematic demonstration of sulfur ylides moiety and its synergistic anti-fouling and selective bactericidal performance [60]. (D) The chemical structures of recent developed zwitterionic crosslinkers [62,63] and the mechanical properties of “complete zwitterionic” hydrogel in compared with that of marketed crosslinker, [65].

Recent progress in zwitterionic hydrogels exhibited a diverse array of derivatives, which can be classified into two categories: zwitterionic monomers and zwitterionic crosslinkers. These derivatives play a pivotal role in extending the functionalities and applications of tough zwitterionic hydrogels. Zwitterionic monomers, through their distinct chemical structures, provide a versatile platform for physical interaction. For example, sulfobetaine vinylimidazole (SBVI) was first introduced by Jiang and colleagues in 2010 [56]. The imidazole-based zwitterionic monomers have tunable solubility in a wide range of ionic strength due to the salt-induced conformation changes. Owing to the unique feature, linear SBVI polymer has been exploited to facilitate ionic complexation with ionic liquid, exhibiting self-healing and fatigue resilience capability (Fig. 4A) [57]. Later on, a novel class of monomer, termed as 3-(1-(4-vinylbenzyl)-1H-imidazole-3-ium-3-yl)propane-1-sulfonate (VBIPS), obtained one benzene ring adjacent to the imidazole moiety. The replacement of methacrylate motifs not only reduces the water retention capability of hydrogel but also provides additional π- π stacking interactions and enhances stretchability and elastic deformability. Trimethylamine N-oxide monomer (TMAO) is another newly developed zwitterionic monomer class (Fig. 4B). Having carbon spacer length (CSL) = 0, TMAO was expected to obtain the strongest hydration layer, surpassing the anti-fouling performance of other betaine motifs [58]. Since resistance to non-specific adsorption is dependent on the hydration, ionic strength can inflict substantial challenges to polyzwitterions in maintaining their anti-fouling performance. Hence, TMAO with a smaller dipole moment combined with an enhanced ordered water layer can mitigate ion-pairing effects, rendering stable fouling resistance in seawater [58,59]. With the same approach, sulfur ylides motif with zero carbon spacer length (CSL) was recently synthesized with unique selective bactericidal and fouling resistance properties [60]. Though the hydration dynamics and surface charge of ylide molecules have not been clearly elucidated, the positively charged ylide molecules might play a role in bacterial membrane disruption (Fig. 4C).

Recent developments in zwitterionic hydrogel have also depicted a surge in zwitterionic crosslinkers toward replacing commercial counterparts as shown in Fig. 4D [[61], [62], [63]]. In general, zwitterionic crosslinkers can contribute to the structural integrity and hydrogel mechanical strength through crosslinking homogenization due to their superior solubility, which surpasses crosslinker content use (>20 mol%). Furthermore, the zwitterionic moieties render these crosslinkers with enhanced wetting and biocompatible properties. Considering that dipole-dipole interactions play a pivotal role in stress dissipation of zwitterionic hydrogels, these crosslinkers not only can induce a “complete zwitterionic” effect in polymeric networks by removing any potential binding sites for bio-foulants, but also enhance intrinsic association between polymer chains for enhance mechanical performance (Fig. 4D).

## 3 Design rationale in synthesis and fabrication

### 3.1 Synthesis approaches

The modulation of mechanical parameters, including polymer density, elastic modulus, fracture stress, and strain, is important in dictating how hydrogels can be implemented in biomedical applications. To this end, they require further adjustment to match their mechanical criteria. The strategies to synthesize tough hydrogels have been generally acknowledged by molecular engineering, crosslinking homogenization and inter- and intra-chain friction enhancement for effective force dissipation [66]. The strategies for synthesizing tough zwitterionic hydrogel and their respective advantages and disadvantages can be summarized as shown in Table 1.

**Table 1:** Various synthesis techniques of tough zwitterionic hydrogels and their advantages and disadvantages.

Synthesis strategies	Representative schemes	Advantages	Disadvantages	Ref.
Supramolecular interactions		- Simplicity of synthesis. - Self-healing capability under certain stimuli. - Antifatigue ability. - Injectability.	- Reduced mechanical properties in polar solvents. - Trade-off between electrolyte swelling and mechanical property (elastic modulus ∼ 1–30 kPa)	[23,[67], [68], [69], [70], [71], [72], [73]]
Nanocomposites		- Simplicity of synthesis. - Multifunctional crosslinking points. - High stretchability (tensile strain reached up to 350 %)	- Agglomeration, which may happen and affect the mechanical properties.	[[74], [75], [76]]
Double/Multiple interpenetrating network		- Excellent strength and fatigue resistance. - Enhanced swelling/deswelling response. - Modulable stiffness (elastic modulus 0.1–0.6 MPa)	- Requirement of large amounts of materials. - Remaining of unreacted monomers and crosslinkers to compromise biocompatibility.	[[77], [78], [79]]
Fiber reinforcement		- Synergistic enhancement of mechanical properties. - Interplay of fluids, fibers, matrix, and interface. - Excellent stretchability (tensile strain 300 % to 2000 %) - Promotion of cell attachment, growth, and migration. - Controllable porosity.	- Requirement of special instruments to prepare the nanofiber. - Difficulty in coating.	[80,81]

#### 3.1.1 Supramolecular hydrogels

Supramolecular interactions offer a promising approach to prevent radical polymerization and mitigate the effects of oxygen inhibition on the polymerization process. Supramolecular hydrogels, characterized by non-covalent interactions such as hydrogen bonding, electrostatic interaction, or host-guest interactions, exhibit distinctive properties including self-healing and responsiveness to environmental stimuli. Specifically, incorporating coupling moieties such as disulfide, imine, or alkyne groups can be achieved through copolymerization techniques. A block polymer consists of two or more distinct blocks of monomers, typically arranged in AB, ABA, or ABC conformations. In supramolecular hydrogels, these blocks can be tailored to impart specific functionalities, such as hydrophilic, hydrophobic, or stimuli-responsive characteristics [82,83]. Recently, ABA triblock copolymers, where A blocks contain SB moieties, have been used for injectable hydrogels [84,85]. Owing to dipole–dipole physical interaction, the polymer can form a self-healable hydrogel, exhibiting improved mechanical durability for long-acting performance. Alternatively, zwitterionic materials can be easily fabricated by mixing hydrophilic polycarboxylic acid with betaine ions, eliminating the requirement for zwitterionic monomers in the synthesis process [86]. Under mechanical stretches, different degrees of physical interactions (betaine-betaine < betaine-polymer < polymer-polymer) induce effective energy dissipation to prevent complete breakage of hydrogels.

#### 3.1.2 Nanocomposite hydrogels

Nanoparticle-reinforced hydrogels combine the merits of nano-scale materials within microscopic polymeric networks. In a typical experimental procedure, the nanoparticles, often inorganic nanoclays, are meticulously dispersed within a hydrogel precursor in an aqueous medium [87]. Following the dispersion, a thermal initiation process triggers radical polymerization from the surface of the nanoparticles. The process effectively crosslinks the polymer chains with the clay sheets and enhances the overall toughening mechanism through uniform crosslinking, which differs from traditional radical polymerization methods. Additionally, polymer ends physically attached to nanoparticle surfaces are only temporarily disrupted under stress, enhancing the durability of the whole network. Aside from mechanical properties, nanoparticles with intrinsic therapeutic effects can further improve the bioactive properties of hydrogels, including anti-microbial properties, anti-inflammation, and anti-oxidation [75,88]. Particularly, the combination of SB-based hydrogels and conductive nanoparticles (graphene and metal nanoparticles) has been exploited in wearable devices [89,90].

#### 3.1.3 Double/multiple interpenetrating networks

Over the past two decades, from their first appearance [91], interpenetrating networks have been utilized to advance the application of hydrogels in biomedical fields. From a mechanistic perspective, these approaches introduce a loosely-crosslinked and ductile second network for effective stress relaxation, while the densely crosslinked and brittle first network acts a sacrificial framework for stress dissipation [92]. The advantage of an interpenetrating network is mechanical performance can be easily tuned via modulating monomer concentration and chemical crosslinkers of precursor reactions. For instance, modulation of crosslinker and monomer composition in the first and second networks can adjust hydrogel mechanical strength, rigidity, flexibility, and water absorption characteristics. This tunability offers the feasibility of customizing hydrogel mechanical properties, rendering it well-suited for any specific application as medical implants [93]. Nevertheless, the first polyelectrolyte network consists of fouling materials that can compromise long-term anti-fouling performance of interpenetrating hydrogels. Furthermore, the synthesis of fully-covered zwitterionic hydrogel was difficult because zwitterionic motifs often exhibit an anti-polyelectrolyte effect, which impedes the precursor solution of the second network from penetrating into the first network and consequently reduces energy dissipation of the whole hydrogel. To mitigate the problem, Jiang's group applied TMAO monomer in the first network, which was able to engulf a large amount of SB monomer for preparation of triple-network hydrogels [79].

The incorporation of natural polymers with synthetic components can provide several benefits, especially in the fabrication of double-network hydrogels [78,94,95]. Owing to their abundance of physical interactions, naturally derived hydrogels capable of stimuli-responsive gelation have been formulated with a primary physical network. This primary network then encapsulates the precursor gel solution of a secondary network. From this setup, the advantage of using a biopolymer in a hybrid system is to provide dynamic bonds for the network. Under repetitive stress, the multiple dynamic crosslinking points within the sacrificial scaffold can break and reform, thereby protecting covalent linkages and preventing complete network failure. A notable example is the microgel-reinforced (MR) system, which has recently been developed to enhance the durability of hydrogels in coating applications [96]. According to the study on MR hydrogels first published by Gong and colleagues in 2018 [97], improving the interfacial contact area is critical in enhancing hydrogel toughness against peeling strength. Because double network hydrogel fabrication is a multiple-step process, microgel-reinforced systems enable relatively easy operation and extended versatility.

#### 3.1.4 Fiber reinforcement

Fiber-reinforced hydrogels integrate the properties of hydrogels with the mechanical robustness and structural architecture afforded by incorporated fibers. While double network hydrogels and fiber-reinforced hydrogels both promote stress dissipation within the network, the critical difference is that fiber reinforcement provides mechanical strength for hydrogels without losing the water absorbency [98]. In addition, fiber-reinforced hydrogels offer the advantage of potential anisotropy, as fibers can be aligned to mimic natural tissues [99], whereas double network hydrogels generally exhibit isotropic properties [100]. Accordingly, the hydrogels provide a high degree of customization through fiber type, orientation, and density, making them particularly suitable for applications requiring anisotropic properties or mimicking fibrous tissues [101]. The primary challenge in successfully incorporating fibers into polymeric networks is to establish efficient adhesive strength between the two systems in aqueous environments. The swelling hydrophilic polymers at water equilibrium often result in weak interfacial interactions with embedded fibers, particularly with macroscale interwoven fibers [102]. This leads to stress mismatch between the polymeric matrix and fibers, resulting in complete fracture of the composite system due to inadequate energy dissipation and crack resistance [103]. Conversely, the unique hydration behavior of zwitterionic hydrogels hold promising toughness enhancement. Specifically, low-crosslinked and long-chain SB hydrogels can dehydrate under elevated mechanical stress, subsequently strengthening their intrinsic dipole-dipole attraction and mechanical interlocking with electrospun polyurethane scaffolds [104]. In a different approach, several studies have extensively utilized nanocellulose fibers to improve the hydrophilicity, homogeneous suspension, and surface area of the fillers [[105], [106], [107]]. These nanofibers can also form physical crosslinks with multiple components of the SB precursor solution, thereby enhancing energy dissipation within the network, but with a trade-off of pull-out strength [107]. Furthermore, the natural/synthetic hybrids also offer simplicity in tough hydrogel fabrication since they are prepared in one-step mixing and can be further implemented in 3D/4D printing to form complex structural variations [108].

#### 3.1.5 Other methods

Other synthesis approaches of zwitterionic hydrogels harnessing dynamic covalent bonds include click chemistry [[69], [70], [71]], coordination complexation [[46], [47], [48], [49], [50]], disulfide bonds [63], boronic ester [109] and dopamine-triggered polymerization [23,72]. These dynamic crosslinks merge the gel stability of covalent crosslinks with the adaptability of supramolecular systems. Thus, they often highly resemble the viscoelastic property that is often observed in biological tissues [110]. When these crosslinks are implemented in zwitterionic systems, they enable hydrogels with tuneable shrinkage or swelling to accommodate cell growth and movement. In addition, the reversible crosslinks offer controlled degradability to ensure proper tissue formation and eventual scaffold resorption, subsequently creating biomimetic microenvironments for successful tissue regeneration, organ repair and cell therapy.

### 3.2 Manufacturing process for zwitterionic hydrogels

The challenge in tough hydrogel fabrication lies between balancing mechanical strength and ease of processing. Recent advancements have focused on optimizing their structure through meticulous adjustments in hydrogel synthesis, such as multi-step crosslinking (interpenetrating network) and soaking (supramolecular hydrogels). However, the start-to-end products are often time-consuming, significantly impeding their application in situ scenarios that demand rapid gelation. On the other hand, maintaining consistency in mechanical properties and hydrogel performance among different production batches is another challenge. Hence, this section will introduce recent approaches to overcome obstacles in hydrogel fabrications toward biomedical implementation.

#### 3.2.1 Manufacturing for bulk hydrogel

**Fig. 5:**
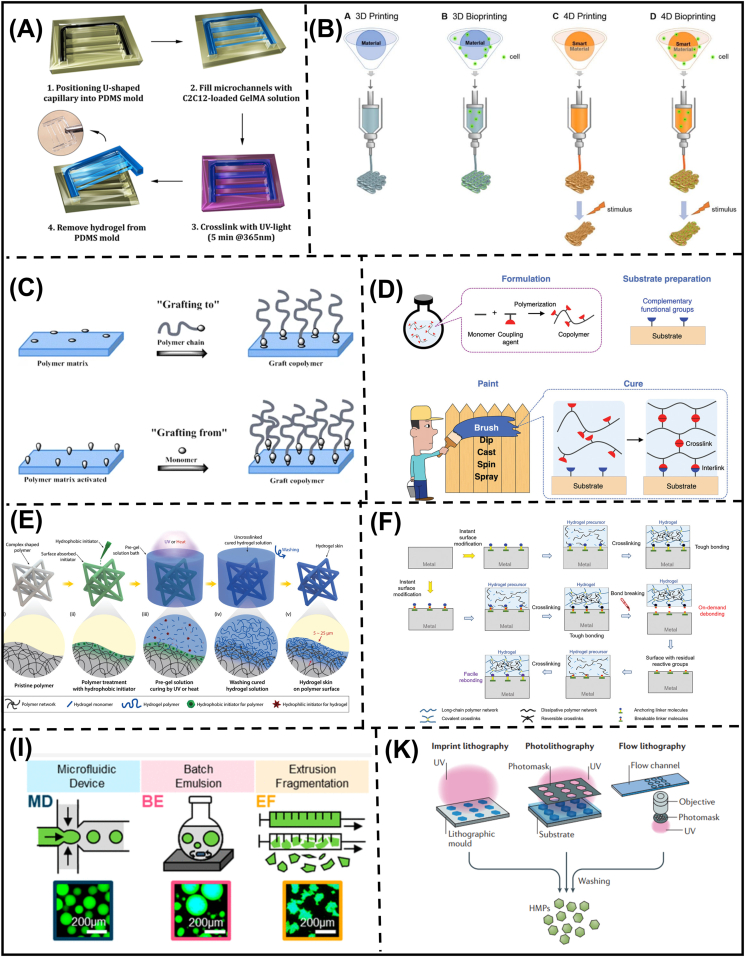
**Various fabrication techniques for macroscopic, coating and micro-scale manufacturing of zwitterionic hydrogels** (A) Schematic illustration of hydrogel molding [111]. (B) Schematic illustration of 3D and 4D printing [119]. (C) Schematic illustration of polymer coating methods [134]. (D) Schematic illustration of hydrogel paint containing copolymers of coupling residues and monomers of interest [125]. (E) Schematic illustration of hydrogel skin procedures on polymer with arbitrary shapes [126]. (F) Design strategy for tough bonding, on-demand debonding, and facile rebonding of hydrogels to diverse metal surfaces [127]. Schematic illustration microgel fabrication using (I) microfluidic device, batch emulsion, fragmentation [135] and (K) lithographic techniques [136].

Molding is a straightforward, cost-effective technique for fabricating hydrogels [111]. The method employs a mold, typically made from materials like polydimethylsiloxane (PDMS), poly(methyl methacrylate) (PMMA), polytetrafluoroethylene (PTFE), or glass [[112], [113], [114]], to shape the hydrogel precursor, which is then cross-linked to conform to the negative shape of the mold (Fig. 5A). The mechanical performance of hydrogels can be tuned by the degree of hydrophobicity of the mold surface, affecting the homogeneity of the network structure [115]. While molding is a simple approach, it can be limited in achieving intricate or highly complex structures. This has led to the development of more advanced 3D and 4D printing techniques. 3D patterning involves layer-by-layer deposition of hydrogel inks to form static structures, while 4D printing harnesses the stimuli-responsive properties of hydrogels to create dynamic, morphing shapes, as shown in Fig. 5B [116,117]. These 3D/4D printing methods overcome the mold dependency of traditional techniques by enabling the fabrication of sophisticated tissue/organ-mimicking structures. However, they often require special hydrogel precursors with self-healing and injectable capability, such as biopolymer/synthetic hybrids and supramolecular polymers [118,119]. Additionally, the reliance on high-end equipment and complex setups restricts the clinical translation of these patterning techniques, limiting their use primarily to laboratory-scale research. In summary, while molding remains a simple and cost-effective hydrogel fabrication method, the emergence of advanced 3D and 4D printing techniques has expanded the possibilities for creating complex, dynamic, and biomimetic hydrogel structures, although challenges remain in their clinical implementation.

#### 3.2.2 Hydrogel coating

In the aspect of hydrogel coating, polymeric networks formed on substrates are commonly synthesized through two methods: (i) “grafting onto” involving the modification of substrate or introduction of anchorable moieties for polymers, (ii) “grafting from” involving the securely anchorable initiator to the surface of the substrate (Fig. 5C). The “grafting from” methods is preferable for surface modification since they enable high-density polymer grafting, which ensures the high coverage of immobilized polymers [120]. In addition, the approach can avoid tedious purification steps of as-prepared polymer, thus aiding in reducing production costs. Regarding fabrication process, two important parameters in hydrogel coatings are the interfacial toughness of anchorable sites and bulk properties of the hydrogel. While the mechanical reinforcement approaches have been extensively summarized and discussed previously [66,121,122], the interfacial toughness between adherents are dependent on the non-specific interactions (Van der Waals forces, electrostatic attractions, hydrogen bonding and hydrophobic associations) and specific interactions (commonly demonstrated as covalent linkages). A greater specificity of interfacial bonds between adherents are highly desirable in hydrogel coating since they confer higher binding strength and stability [123].

Silane coupling agents are widely used for the surface modification of inorganic and silicon-based materials, incorporating various functional groups onto decorated surfaces, including amine, thiol, azide, and vinyl groups [124]. Due to the commercial availability, multiple studies have combined silane chemistry for hydrogel coating. Suo and colleagues introduced an approach that utilizes a coupling agent (3-(trimethoxysilyl)propyl methacrylate) (TMSPMA) to facilitate silane groups with hydrophilic copolymer (Fig. 5D) [125]. Considering that open-air polymerization may impede chain propagation efficacy, the “grafting onto” strategy renders large-surface modification via various techniques, including dip coating, spin coating, casting, and spraying. For organic substrates like elastomers and hydrophobic polymers, hydrogel coating can be realized through mechanical interlocking (Fig. 5E) [126]. In brief, before hydrogel synthesis, porous polymeric materials are soaked with hydrophobic initiators, which further penetrate and form a thin segment on the outer portion of the substrates. Then, viscous hydrogel precursor is soaked on surfaces with hydrophobic initiators for photo-curing. Notably, the oxygen inhibition is restrained on the outermost of hydrogel coating since initiation and propagation stages stem from immobilized initiators, resulting in a thin and conformal gel coating on the substrates.

For metal substrates, it is necessary to provide anchorable sites for hydrogels by the selection of coupling agents, including catechol, thiol, carboxyl and silane. After the deposition of primer layer, hydrogel coating can be deposited via brushing, dip coating, casting, spinning, and spraying (depending on precursor solution viscosity and dipping time of the hydrogels) [125,127]. In particular, Yue and colleagues introduced a linker molecule with a carboxylate group one end and polymerizable acrylamide moieties on the other [127]. The hydrogel coating was prepared by a two-step setup, in which metallic surfaces were first functionalized via the coordination of carboxylate groups. Hydrogel formation can be conducted in the second step through photo-crosslinking with vinyl groups. To broaden the applicability of the approach, the group continued to incorporate disulfide linkages within the backbone of linker molecules. This enables on-demand debonding via GSH treatment and re-bonding via thiol-ene addition mechanism to form thioether linkages, offering universality and versatility for hydrogel coatings (Fig. 5F).

#### 3.2.3 Manufacturing for microscale hydrogels

Microgels have gained widespread attention due to their numerous advantages, finding applications in diverse fields such as cell culture, therapeutic delivery, bioprinting, and tissue engineering [128,129]. Various methods have been developed to fabricate microgels for these applications, including microfluidics, emulsion polymerization, and mechanical disintegration (Fig. 5G). Among these approaches, microgels can be fabricated in microfluidic devices with high spatial precision by generating aqueous hydrogel precursor droplets within a continuous oil phase. The droplet size is influenced by the flow rate, interfacial tension, and device geometry, allowing for size variations of 5–1000 μm with 1–2 % dispersity. However, microfluidic production rates are usually lower than alternative techniques [130]. Batch emulsion is an alternative approach, where an aqueous hydrogel precursor is introduced into a continuous oil phase and stirred to generate cross-linked droplets. Emulsion fabrication offers faster production rates and flexibility in modulating droplet size but exhibits greater size variations compared to microfluidics. In a single batch, droplet diameters may exhibit an order of magnitude difference, ranging from approximately 1 to 1000 μm [131]. Finally, mechanical fragmentation is a simple, affordable, and scalable method that does not require oil and surfactants. The average microgel size and porosity can be controlled by adjusting the mesh size, and the resulting granular hydrogel supports long-term cell culture due to its higher mechanical stability. The main disadvantage is the production of irregular shapes that may hamper injectability and dosing accuracy for drug delivery. Beyond lab-scale preparation, microgels can also be prepared through lithographic and photolithographic strategies (Fig. 5H). Lithographic methods for fabricating non-spherical microgels offer precise control over particle shape and size, which are best suited for complex 3D structures with specialized molds [132]. On the other hand, photolithography provides excellent resolution for small features but is often limited to 2D shapes [131]. These techniques allow for high precision, reproducibility, and potential for large-scale production [133]. However, they may face challenges in practice due to their dependence on specialized equipment and tedious calibration.

## 4 Implementation of zwitterionic hydrogels in biomedical applications

### 4.1 Medical coatings

Biofouling is a persistent challenge in the biomedical field, jeopardizing the efficacy and safety of various medical devices and implants. This issue, marked by the unwanted accumulation of biological foulants such as proteins, cells, and microorganisms on surfaces, can lead to device failure, increased infection risks, and compromised therapeutic outcomes. As medical technology advances, the demand for effective anti-fouling strategies has become increasingly critical. From implantable devices to diagnostic tools, the pursuit of surfaces that resist biological contamination is driving innovation in materials science and bioengineering. In this context, zwitterionic hydrogel coatings offer multiple benefits: they prevent the initial attachment of bacteria, enhance patient comfort, and reduce inflammation and injury at implant sites (Table 2).

**Table 2:** Recent advances of zwitterionic hydrogels in anti-fouling coating.

Zwitterion	Source	Crosslinking method	Coating chemistry	Significant outcomes	Ref.
Sulfobetaine	Synthetic	Schiff base and Michael addition	Catechol chemistry	∗ Anti-fouling in ex vivo and blood-circulating systems for 2 h	137
Carboxybetaine	Synthetic	Schiff base and Michael addition	Catechol chemistry	∗ Dual function: anti-fouling and contact killing ∗ Resistance of biofilm formation in bacteria-contacting environment over 30 days.	138
Sulfobetaine	Semisynthetic	Disulfide bonds	Catechol chemistry	∗ Functionalizable coating ∗ Anti-fouling for 7 days in buffer shearing	139
Carboxybetaine, sulfobetaine	Synthetic	Covalent crosslinker	Mechanical interlock	∗ Improve mechanical strength and adhesion via microgel-reinforced method ∗ Anti-fouling for 21 days in buffer shearing	142
Sulfobetaine	Semisynthetic	Covalent crosslinker	Mechanical interlock	∗ Dual functions: anti-fouling and pH-responsive antibacterial activities	145
Carboxybetaine	Synthetic	Covalent crosslinker	Silane chemistry	∗ Prevention of bacterial attachment over 14 days	146
Sulfobetaine	Synthetic	Covalent crosslinker	Catechol chemistry	∗ pH-responsive antibacterial activities	149
Sulfobetaine	Synthetic	Covalent crosslinker	Catechol chemistry	∗ Swell and deformation resistance in water and saline solution	150
Phosphobetaine	Synthetic	Covalent crosslinker	Mechanical interlock	∗ Improved hydrophilicity of the silicone rubber and reduced the deposition of bovine serum albumin ∗ Release of Zn2+ for killing bacteria	151
Sulfobetaine	Semisynthetic	Covalent crosslinker	Catechol chemistry	∗ Robust stability but also excellent biocompatibility and safety ∗ Antiadhesion of proteins, cells, platelets, bacteria, and anti-thrombosis	152

**Fig. 6:**
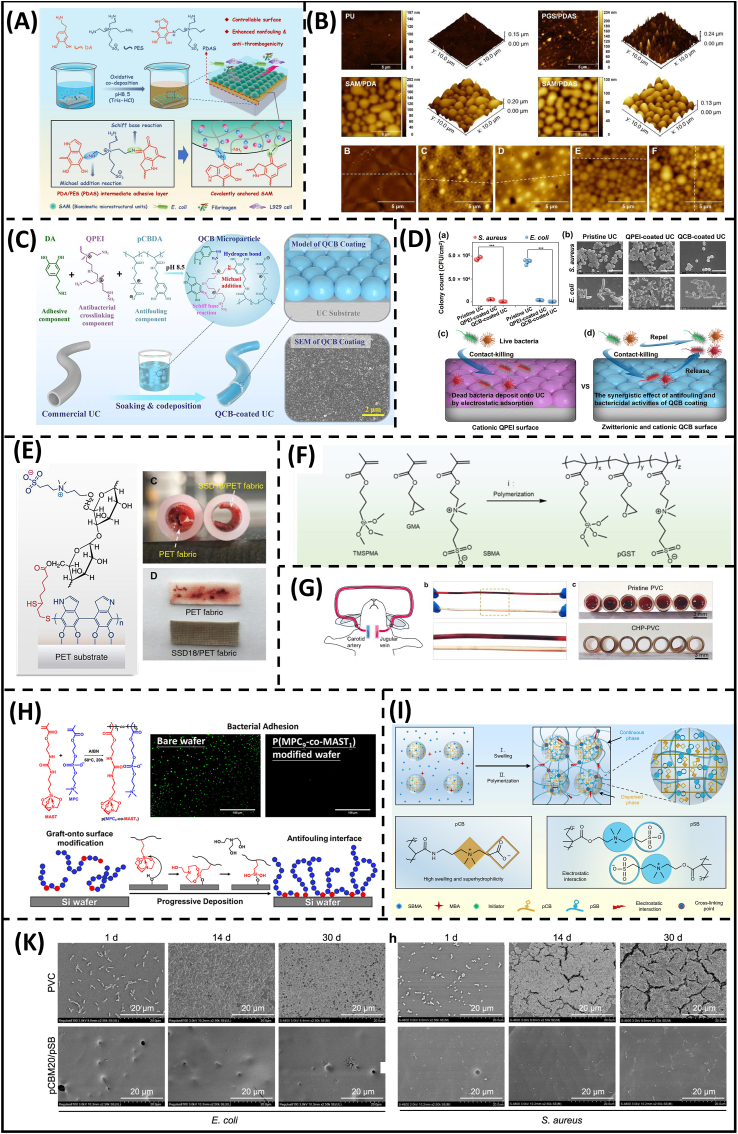
**State-of-the-art zwitterionic hydrogels in medical coating** (A) Schematic illustration of the codeposition of microgels and polydopamine (SAM/PDAS) for anti-fouling surface. (B) Surface morphology of different coating conditions to emphasize the hierarchical control over surface modification (from nanoscale polydopamine particles to microscale hydrogels) [137]. (C) Nanogel formation from supramolecular crosslinking between catechol-functionalized polymer and polydopamine particle. (D) Synergistic kill-and-release performance of the coated urinary catheters after coincubation with bacterial solution for 14 days [138]. (E) Schematic illustration of SB-ST-D hydrogel coating on PET substrates. Anti-thrombogenicity of the SB-ST-D hydrogel coating on PET fabric [139]. (F) Design principles and procedures of composite hydrogel paints. (G) Antithrombosis of hydrogel paint in the rabbit models [140]. (H) Schematic illustration of novel polymerizable silatrane (MAST) in anti-fouling coatings [141]. (I) Schematic illustration of complete zwitterionic microgel-reinforced hydrogel coating and (K) SEM images of hydrogel coatings after coincubation with bacterial solution for different period of time [142].

The surface independency of mussel-inspired chemistry offers a versatile platform for hydrogel coatings. Notably, the strategy offers post-modification strategies through mild reaction conditions (room temperature, weak base aqueous solvent, and oxygen tolerance). Harnessing these advantages, SB and CB motifs have been implemented for a one-step surface zwitterionization. In particular, a precursor containing betainized polyethylenimine/polydopamine (PEI/PDA) and zwitterionic microgels has been prepared for non-fouling surfaces, as demonstrated in Fig. 6A [137]. Controlling surface zwitterionization from molecular scale to microscale can completely prevent foulant penetration through exposed gaps or unreacted sites on PDA nanoparticles (Fig. 6B). It is worth noting that the hydrophilic betainized PEI/PDA deposition has poor coating efficiency compared with PDA alone, making its stability insufficient under prolonged hemodynamic shear stress. To this end, microgel deposition further supplements adhesive strength, preventing coating detachment and maintaining their hydrophilicity for 14 days under PBS and whole-blood incubation. Nevertheless, microgel implementation could increase the viscosity of coating solutions, limiting their use in enclosed tube-shaped surfaces. Accordingly, the technique was further improved by Liu's group to enable surface modification on curved materials like urinary catheters via a simple soaking method (Fig. 6C) [138]. Specifically, nanogels were formed on coating surfaces through crosslinking reactions between linear catechol-functionalized CB copolymers and quaternized PEI/PDA nanoparticles. In addition, the kill-and-release synergy of nanogel coating provides excellent resistance to bacterial fouling for 14 days (Fig. 6D).

Integrating polysaccharides with zwitterionic motifs marks an effort to overcome the bio-inertness of zwitterionic hydrogel. One of the key advantages of polysaccharides is their ease of modification, allowing for the introduction of functional groups that can further enhance biological interactions. In addition, polysaccharides can introduce multiple bioactive properties to hydrogel coatings. Particularly, chitosan, well-known for its antibacterial capability, was coupled with PC motifs and photo-crosslinked via glycidyl methacrylate to functionalize polyethylene terephthalate (PET) substrates, providing synergistic effects to combat bacterial transfection [143]. Hyaluronic acid, a key extracellular matrix component, is well-known for multiple therapeutic effects, including antioxidation, anti-inflammation, and anti-apoptosis [144]. Harnessing the susceptibility against enzyme degradation of the biopolymer, Zhang and colleagues have integrated SB motifs with methacrylate-functionalized hyaluronic acid to produce a “self-defensive” hydrogel coating [145]. Bacterial accumulation can compromise the anti-fouling performance of SB. Hyaluronidase secreted by the bacterial colony can dissolve a portion at the adhesive site, reducing the infection threat for medical devices. In another approach, starch was used as cell-friendly platform for coating permanent medical implants such as vascular grafts, as shown in Fig. 6E [139]. Interestingly, available coupling sites on the biopolymer can be supplemented with REDV peptide (a sequence of Arginine-Glutamic Acid-Aspartic Acid-Valine) to promote endothelial layer formation in blood-contacting sites. This ensures the long-lasting action of hydrogel coatings on medical implants.

Silane chemistry showed its implementation in hydrogel coatings. In particular, surface zwitterionization of crosslinked polymer was performed by Chen and colleagues [146]. The authors presented a hydrogel coating with anti-fouling regeneration capability. CB ester analogs in the outermost layer can be hydrolyzed to form novel zwitterionic moieties. However, the previous study did not address the interfacial adhesive strength of the hydrogels. To close this gap, Li and colleagues introduced a composite hydrogel paint consisting of copolymers and microgel components (Fig. 6F and G) [140]. In brief, linear copolymers containing silane residues were allowed to penetrate the microgel network for enhanced force dissipation. Furthermore, additional coupling sites between copolymers and microgels were provided, promoting chain density and interconnection between different components in hydrogel paint. As a result, the coating exhibited anti-fouling performance for 14 days. In another approach, Huang and colleagues aimed to improve stability and silanization reaction through copolymerization PC monomers with polymerizable silatrane molecules (Fig. 6H) [141]. Silatrane is more superior to silane in multiple aspects, including hydrolytic resistance and controlled silanization. Moreover, the surface functionalization using silatrane also exhibits ten times faster in coupling rate [147]. Accordingly, methylacrylate silatrane linker offers ease of operation for both “grafting from” and “grafting onto” polymerization of PC monomers while remaining structurally stable in moist condition (10 v/v% of water) for 5 days.

Benzophenone, a hydrophobic initiator, has been applied extensively as an anchorable linker for zwitterion immobilization on porous polymeric materials and elastomers. Very recently, benzoylphenyl acrylate, a polymerizable form of benzophenone, has been implemented as a primer layer onto elastomer [148]. In their study, the analog acted as both crosslinkers and initiators, forming a stable anchoring platform with anti-drying and anti-swelling capability owing to the tight mechanical interlock via hydrophobic association. Moreover, the surface zwitterionization technique can be further implemented using “grafting from” approach. Further exploration of benzophenone-mediated coating, Li's group has implemented the MR gel technique in a “complete zwitterionic” system, as shown in Fig. 6I [142]. In this work, the CB microgels were first prepared and then immersed with SB hydrogel precursor to form the final hydrogel. The strategy enables more energy transmission from CB to SB chains while preventing debonding and the total breakage of the microgels under peeling, exhibiting excellent fouling resistance after 30 days co-culturing with bacterial solution (Fig. 6K). Hence, the strategy provides a solution for industrial-scale production, enabling post-functionalization platforms to expand the hydrogel coatings in biomedical applications.

### 4.2 Medical implants and wearable devices

In this section, we address the challenges of hydrogel fabrication for medical implants and wearable devices, which are critical for the successful development and application of these technologies. In this field, the hydrogel design is exclusively focused on the bulk effect of the materials and their stability in response to external conditions. Consequently, this aims to replace or improve existing medical devices. These designs, including sources of zwitterionic materials, the hydrogel synthesis approaches and their achievement in mechanical performance, are summarized in Table 3.

**Table 3:** Recent advances of zwitterionic hydrogels in medical implants.

Zwitterion	Source	Crosslinking method	Mechanical reinforcement technique	Significant mechanical properties	Significant outcomes	Ref.
Sulfobetaine vinylimidazole	Synthetic	Covalent crosslinkers	Interpenetrating network	Tensile strength: 500–600 kPa Young modulus: ∼100 kPa	∗ Kill-and-release mechanism	153
Lysine acrylamide, sulfobetaine	Synthetic	Covalent crosslinkers	Interpenetrating network	Tensile strength: 1073 ± 153 kPa, Young's modulus: 369 ± 9	∗ Superior anti-fouling in vitro circulation of bacteria solution	154
Carboxybetaine, sulfobetaine	Synthetic	Covalent crosslinkers	Interpenetrating network	Compressive strength: 22.3 MPa Young modulus: 0.1–1 MPa	∗ Implantability for 1 year	155
Sulfobetaine	Synthetic	Dual crosslinks (covalent and multiple physical bonds)	Dopamine-triggered hydrogel	Tensile strain: 350 %	∗ High mechanical flexibility and gauge factor at 0.71	72
Sulfobetaine	Synthetic	Dual crosslinks (covalent and hydrophobic interaction)	Supramolecular hydrogel	Maximum tensile strain: 157.5 %	∗ High sensitivity under low dehydration ∗ Swelling resistance and stable strain sensitivity after being soaked in water for 2 month	157
Sulfobetaine	Semisynthetic	Physical interactions (hydrogen bonds and electrostatic interaction)	Supramolecular hydrogel	Maximum tensile strain: 1137 %	∗ Gauge factor at 1.25 ∗ Dual-modal strain and temperature sensor over a wide temperature range (-40–80 °C)	106
Proline, sulfobetaine	Semisynthetic	Dual crosslinks (covalent and electrostatic interaction)	Supramolecular hydrogel	Compressive modulus over 0.1 MPa	∗ Complete preservation of mechanical flexibility at −40 °C	158

**Fig. 7:**
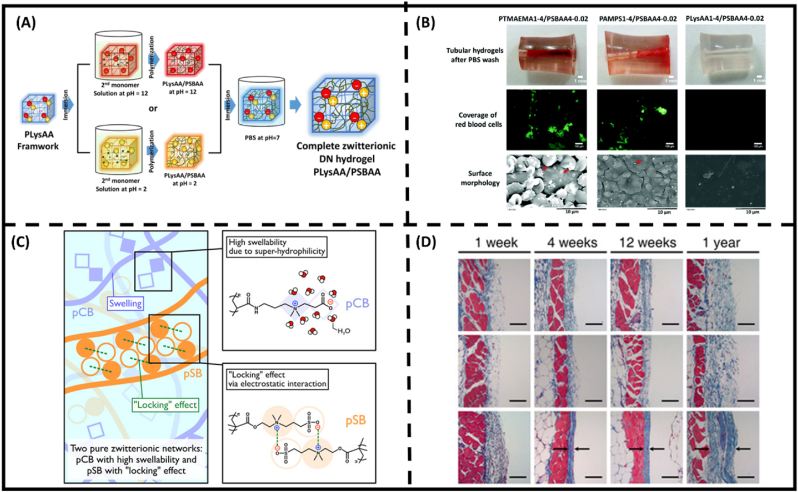
**State-of-the-art zwitterionic hydrogels in medical implants utilizing zwitterionic interpenetrating network approaches.** (A) Schematic illustration of the synthesis of a complete zwitterionic DN hydrogel using the pH-responsive pLys framework [153,154]. (B) The resulting image of tubular DN hydrogels was obtained after whole blood circulation for 2 h. The fluorescence image showed the red blood cell distribution on tubular DN hydrogels, while the SEM image depicted the surface morphology of the tubular hydrogels. Red arrows pointed to the presence of platelets [154]. (C) Schematic illustration of the design principles of the high-strength poly(carboxybetaine) (pCB)/poly(sulfobetaine) (pSB) ZEN hydrogel [155]. (D) Skin tissues with different hydrogel samples underwent Masson's trichrome staining after implantation for 1, 4, and 12 weeks, and 1 year [155]. (For interpretation of the references to colour in this figure legend, the reader is referred to the Web version of this article.)

For medical implants, various approaches utilizing double-network hydrogels have been presented [79,[153], [154], [155]]. In particular, Huang and colleagues incorporate highly salt-responsive pSBVI with quaternary amine-based polyelectrolyte as the first network [153]. As a result, the materials exhibited a swift surface switching between contact-killing mechanism (stemmed from quaternary amine) and passive anti-fouling performance of SBVI moieties, ensuring long-term prevention against nosocomial infection. Blood clotting and thrombosis are significant challenges in engineering tough medical implant hydrogels. Accordingly, fully zwitterionic hydrogels are considered the most promising design since they eliminate the potential platelet binding sites. Harnessing the unique swelling behavior of amino acid-derived zwitterion, Huang's group further fabricated a double-network hydrogel composed of Lys/SB system. Under low pH conditions, Lys can be temporarily shifted to polyelectrolyte conformation, which showed high absorption to precursor solution of zwitterionic monomers to induce a second network (Fig. 7A) [154]. Furthermore, the material can be implanted in 30 days without causing a foreign body effect (Fig. 7B). In another approach, CB/SB combination was implemented for double network hydrogel to prolong further anti-fouling performance (Fig. 7C) [155]. From a synthesis perspective, although all zwitterions induce the anti-polyelectrolyte effect, the swelling behavior of the motifs under DI water varies depending on their carbon spacer length (CSL) and dipole moment [156]. In this context, the CB motif can absorb the pre-gel solution SB monomer, ensuring sufficient polymer density in the second network. As a result, the material exhibits resistance to capsule formation for one year (Fig. 7D).

**Fig. 8:**
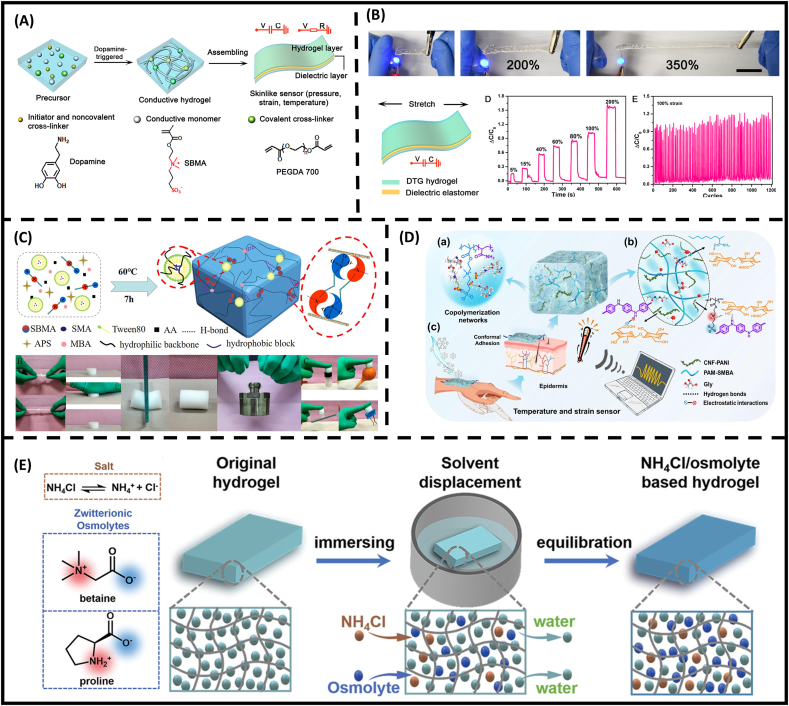
**State-of-the-art zwitterionic hydrogel in wearable sensor.** (A) Design and (B) applications of stretchable, conductive dopamine-triggered gelation (DTG) hydrogels as strain and pressure sensors [72]. (C) Preparation and mechanism diagram of the hydrogel P(AA-SMA-SBMA) with excellent mechanical properties and adhesion adhered to plastic, rubbers, glass and paint tube. (D) The design of zwitterionic (PBA/CPA/Gly) hydrogels with strain and temperature versatile responsiveness [106]. (E) Preparation of antifreeze solutions containing NH_4_Cl in water and molecular structures of betaine and proline.

Catechol chemistry has been widely implemented in hydrogel engineering to provide underwater adhesiveness and highly stretchable ionic-conducting devices. Dopamine molecules are catalyzed by oxygen, which results in a spontaneous chain reaction so-called “autooxidation”. This renders the dopamine-triggered polymerization approach with more oxygen tolerance. In the fabrication perspective, since the synthesis processes do not require heat, UV light and intricate chemical instruments during polymerization, this makes applicability in scale-up manufacturing. The in situ gelation from facile mixing dopamine with acrylate monomers formed tough hydrogel with enhanced stretchability and self-healing capability through multiple physical interactions. In addition, the gels exhibited unique features such as optical transparency and high catechol-functionalization degree (50 wt% in total monomer) in comparison with other post-functionalization approach of dopamine (Fig. 8A) [23,72]. From these merits, the implementation of the material has been expanded to a dual-responsive skin sensor. The exclusive phase transition capability was observed at upper critical solution temperature (UCST) at ∼10 °C, reversibly switching the hydrogel from transparent to opaque at elevated temperature. Furthermore, the soft yet stretchable hydrogel enables high gauge factor (GF) at 0.71 (Fig. 8B) [72]. Regardless of the various benefits of catechol-functionalized hydrogels, the dull reaction rate of dopamine autooxidation can cause a challenge in scaling up the process. Recently, the implementation of dopamine-triggered gelation was introduced to address the issue, holding promises as medical devices and actuators. Recently, Lee and colleagues utilized catechol-functionalized microgels to fabricate tough dual-layer hydrogel [73]. In the study, the strategy facilitates highly potent hydroxyl OH^−^ radicals via a Fenton-like reaction to mitigate the termination of polydopamine and improve monomer conversion efficiency within 120 min of in situ gelation. These approaches could further supplement the expansion of dopamine-triggered gelation for wearable devices.

Wearable devices for mechano-sensing applications utilizing SB motifs can show superior mechanical strength and durability. Yet, mechano-sensitivity can still be compromised by unfavorable environment conditions, causing gel dehydration (or freezing) under hot (or sub-zero) temperatures. To mitigate these drawbacks, Xu and colleagues copolymerized SBMA monomer with hydrophobic octadecyl methacrylate (P(AA-SMA-SBMA)) to prevent further loss of gel flexibility (Fig. 8C) [157]. In another approach, highly conductive yet tough hydrogels composed of polyaniline-grafted TEMPO (2,2,6,6-tetramethylpiperidine-1-oxyl radical)-oxidized cellulose nanofibers within linear SB-containing copolymer have been prepared in a one-pot procedure (Fig. 8D) [106]. Notably, the strong ionic hydration of SB motifs entrapping glycerol (Gly)-water binary solvent can prevent encapsulated water from freezing, preserving gel adhesive strength and fracture strain at 900 % at −40 °C while maintaining strain per resistance signal at linear proportion. Considering that additives such as antifreeze agents and surfactants could cause cytotoxicity, Zhang's group co-loaded proline osmolytes with a conductive component to inhibit hydrogen bonding and water crystallization at −40 °C while preserving the conductivity and mechanical flexibility of the hydrogels (Fig. 8E) [158].

### 4.3 Tissue engineering

3D cell encapsulation has emerged as a crucial aspect of tissue engineering due to several benefits, including tissue mimicry, enhanced cell-cell and cell-environment contact surface and long-term preservation of therapeutic cells. Nevertheless, cell encapsulation is a tedious technique that often produces batch inconsistency. Especially in stem cell culture, the exposure to hydrophobic surfaces, including polystyrene flasks, could lead to their differentiation. To this end, the bio-inertness of zwitterionic hydrogels, in a certain extent, can preserve cellular biochemical metabolism and stemness through preventing non-specific interaction between cell culture and external culture environment. From a mechanical enhancement perspective, zwitterionic hydrogel is required to possess self-healing capability and durability yet obtain adequate chain flexibility to promote long-term cell expansion and functionality preservation. Overall, the current design rationales of zwitterionic hydrogels will be discussed in this section and are summarized in Table 4.

**Table 4:** Recent advances of zwitterionic hydrogels in tissue engineering.

Zwitterion	Source	Crosslinking method	Cell type	Explored properties	Ref.
Carboxybetaine	Synthetic	Strain-promoted azide-alkyne cycloaddition (SPAAC) ‘click’ reaction	Hematopoietic stem and progenitor cells	∗ Degradability ∗ 73-fold increase in long-term hematopoietic stem cell (LT-HSC) frequency	69
Sulfobetaine	Synthetic	Photo-reversible crosslinkers	Adipose-derived stem cell	∗ Self-healing and degradable ∗ Protect stemness ∗ Suppress inflammatory reactions	71
Sulfobetaine	Synthetic	Covalent crosslinker	Adipose-derived stem cell	∗ Bulk feature, shear-thinning, and self-healing properties ∗ Extrusion bioprinting ∗ High level of viability and stemness	161
Carboxybetaine, sulfobetaine	Semisynthetic	Phenolic coupling	Chondrocyte	∗ Self-healing, injectable properties ∗ Biodegradability	162
Sulfobetaine	Semisynthetic	Hydrogen and ionic bonds	Adipose-derived stem cells	∗ Self-healing ∗ Antibacterial	163
Carboxybetaine, sulfobetaine	Semisynthetic	Ionotropic gelation	Rat islet	∗ Improved islet encapsulation ∗ Cell encapsulation therapy for T1D	164
Carboxybetaine	Semisynthetic	Micheal addition	T cell	∗ Biodegradability ∗ In situ gelation	165
Carboxybetaine	Semisynthetic	Disulfide bonds	Human mesenchymal stem cell	∗ Self-healing and degradable properties ∗ Protect stemness ∗ Suppressed inflammatory reactions	166

**Fig. 9:**
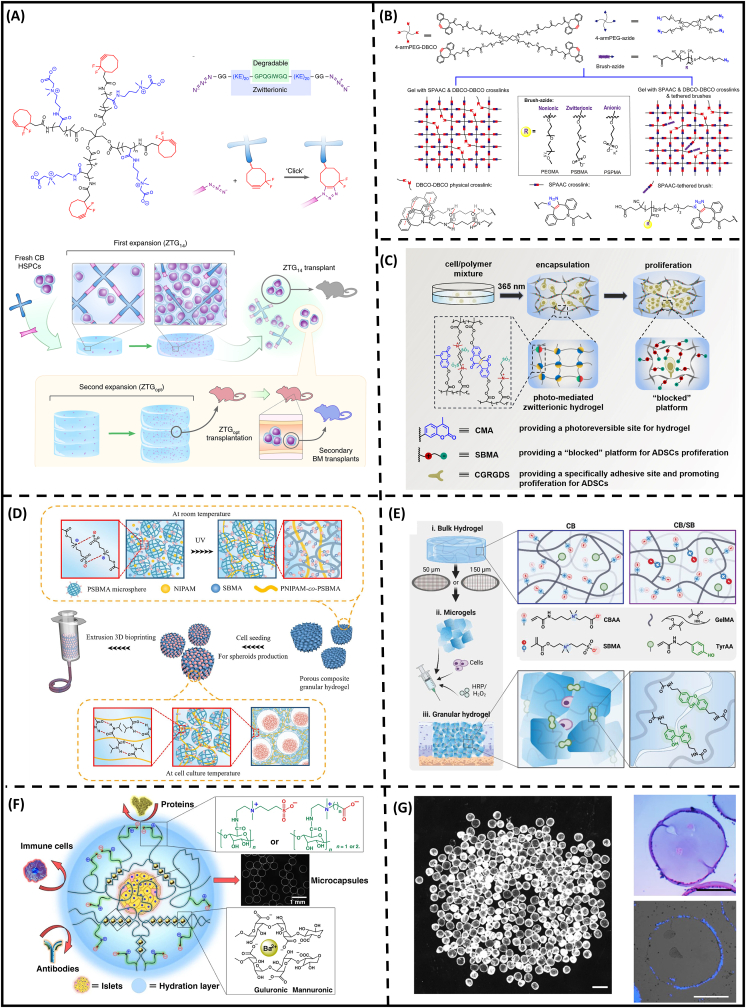
**State-of-the-art zwitterionic hydrogels in tissue engineering utilizing click chemistry.** (A) Schematic illustration of zwitterionic hydrogels formed from star-shaped CB polymers [69]. (B) Depiction of a facile strategy for prospective tuning of hydrogel viscoelasticity and stiffness through the covalent incorporation of azide-terminated polar nonionic (PEGMA), zwitterionic (SBMA), or anionic (SPMA) polymethacrylate brushes [70]. (C) Schematic illustration of the zwitterionic hydrogel as a “blocked” platform for ADSCs expansion and stemness maintaining [71]. (D) Fabrication and function of porous composite granular hydrogel for bioprinting and establishment of in vitro drug screening model [161]. (E) Illustration of zwitterionic granular hydrogel for cartilage tissue engineering [162]. (F) Injectable and self-healing hydrogel (DSC), comprising sulfobetaine-derived dextran and carboxymethyl chitosan to excellent microenvironment for islets. (G) Islets encapsulation of zwitterion-functionalized polysaccharides [163].

Radical polymerization is usually not preferable for cell encapsulation since the polymerization processes must abide by strict requirements, including low cytotoxicity and fast polymerization kinetic. In typical cell encapsulation procedures, the initiator, potassium persulfate (KPS), and accelerator, tetramethylethylenediamine (TEMED), were used to trigger gelation for a few min before cell suspension [159]. Although the methods enable cell entrapment without additional instruments like UV lamps, they may cause uneven cell distribution and changes in cell morphology, polarity, and division. To this end, click chemistry has been utilized extensively to facilitate in situ gelation under mild reacting conditions [[69], [70], [71],^160^]. In particular, Bai and colleagues introduced a four-arm CB end-capped with difluorinated cyclooctynes and bis(azide)-modified degradable polypeptide (Fig. 9A) [69]. 4-armed CB crosslinks exhibited exceptionally fouling resistance, creating a so-called “blank slate” that prohibited background influence on the pluripotency of stem cells, concurrently showing stable cellular expansion regardless of the culture media used. With the stability, the zwitterion-based cellular niches can preserve the stemness for 24 days in comparison with freshly-made cells. The report marks for the first time that zwitterionic hydrogel was used for ex vivo expansion for stem cells. The “blocked” culture concept was further developed by Song's and Li's group with on-demand sol-to-gel transition [70,71]. In these studies, dynamic covalent crosslinks and physical interactions are implemented to tune temporal dissociation of zwitterionic network via external stimuli such as light or mechanical stress (Fig. 9B). For a general demonstration, clickable moieties were incorporated with SB motifs, forming entirely physical hydrogels with dual crosslinking strategies through gel softening and stimuli-responsive toughening (Fig. 9C): (i) SB-mediated “blocked” platform protects stem cells from differentiation, (ii) the dynamic bonds enable temporary breakage, reducing gel stiffness for stem cell expansion. As a result, these approaches open up a novel avenue utilizing stimuli-responsive hydrogels for continuous culture and harvesting of stem cells.

Tough zwitterionic hydrogels are critical for promoting cellular proliferation in load-bearing tissue substitutes, including cartilage and bone tissue regeneration. From the therapeutic perspective, these zwitterionic hydrogels are required to be injectable, which ensures their effective delivery to bone fragments or joint gaps. Due to these reasons, microgel-reinforced hydrogels have emerged as potential candidates for regenerative therapy [161,162]. General preparation began with microgel fabrications for injectable formulation (Fig. 9D), followed by integrating with a second gel network for comprehensive stability of in situ cell encapsulation. It is worth noting that parameters, including microgel content and particle size, are proportionally correlated with interparticle interaction, which plays crucial roles in the injectability or printability of the microgel system. In particular, mechanically-fragmented CB/SB copolymer microgels at a smaller size (150 μm-grid) would provide an appropriate porosity for cell suspension, concurrently promote adequate shear rate, viscosity, and yield strength for therapeutic implementation (Fig. 9E) [162]. Furthermore, the incorporation of zwitterionic motifs is crucial to the functionality of this approach. While SB residues can enhance intermolecular interactions for tough microgel composites, CB groups, with their stronger hydration shells, are more effective in preserving stemness, leading to improved therapeutic outcomes.

From a scale-up standpoint, semi-synthetic hydrogels are much more cost-effective than fully synthetic materials since they require minimal modification and are produced from an abundant and sustainable polysaccharide biomass. Nevertheless, polysaccharides are susceptible to cell adhesion due to available binding sites, leading to the so-called “cell overgrow” effect and fibrosis when exploited in tissue grafting. To overcome the issue, zwitterion-functionalized biopolymers have been recognized in multiple studies for cell encapsulation [[163], [164], [165], [166]]. In particular, encapsulated cells were protected against protein adsorption by strong hydration layers of CB-decorated alginate and hyaluronic acid, avoiding graft rejection and improving hydrogel integration within the host body [[164], [165], [166]]. Aside from anti-fouling performance, a combination of SB-modified dextran and CB-functionalized chitosan was employed in stem cell therapy to alleviate burnt wounds (Fig. 9F and G) [163]. The composite materials not only lessen the host immune response, but also condition extracellular meshwork remodeling at wounded sites by promoting collagen deposition, angiogenesis, and macrophage M2 polarization.

### 4.4 Drug delivery

Zwitterionic hydrogels have been implemented widely in drug delivery systems (DDSs) due to their superior hydrophilic capability. For DDS system criteria, hydrogel-based materials are required to bypass various anatomical, dynamic, and metabolic barriers in the body [167]. To this end, zwitterionic hydrogels have displayed several benefits as follows: (i) masking drug from host immune recognition to increase circulation time, (ii) improving site-specific drug delivery through stimuli-responsive (including pH, ion, and temperature), (iii) encapsulating nanotherapeutic through dynamic interactions, (iv) protecting wound sealant from mechanical damage, and (v) providing additional bio-active capability. To elucidate these points, design considerations for DDSs in synthesis, fabrication, and post-modifications in zwitterionic hydrogels will be discussed in this section and presented in Table 5.

**Table 5:** Recent advances of zwitterionic hydrogels in controllable drug release.

Zwitterion	Source	Crosslinking method	Drug	Encapsulation mechanism	Release mechanism	Ref.
Sulfobetaine	Synthetic	Covalent crosslinkers	Sunitinib and chlorin e6	Physical encapsulation	Redox-responsive	171
Sulfobetaine	Synthetic	Covalent crosslinkers	Curcumin	Hydrophobic interaction	Sustained release	172
Carboxybetaine, sulfobetaine	Synthetic	Covalent crosslinkers	Cationic heme-mimetic gallium porphyrin	Electrostatic interaction	Sustained release	173
Sulfobetaine	Synthetic	Covalent crosslinkers	Levofloxacin	Hydrophobic and intermolecular interactions	Sustained release	174
Sulfobetaine	Synthetic	Covalent crosslinkers	Rifampicin	Micelle-crosslinked into hydrogel	Mechano-responsive drug release	175
Sulfobetaine	Synthetic	Covalent crosslinkers	Cerium oxide nanoparticle tagged with microRNA-146a	Physical encapsulation	Sustained release	176
Sulfobetaine	Semisynthetic	Covalent crosslinkers	Doxorubicin, 1-methyl-d-tryptophan	Hydrophobic interaction	Sustained release	179

**Fig. 10:**
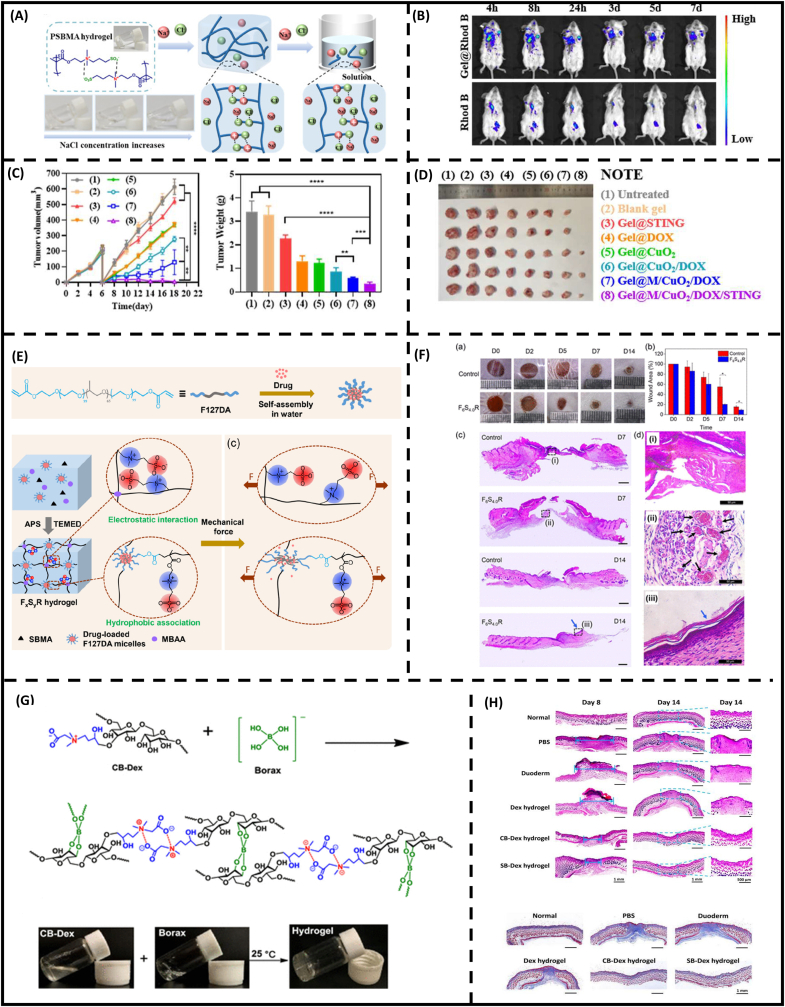
**State-of-the-art zwitterionic hydrogel in drug delivery.** (A) Schematic illustration of pSBMA hydrogel with electrostatic interactions cross-linked network [169]. (B) *In vivo* retention fluorescence image of the Rhod B solution and Gel@Rhod B. (C) Tumor volumes and weights in different groups on day 20. (D) Images of excised tumors on day 20 ^170^. (E) Schematic illustration of Drug-Loaded F127DA Micelle equipped with crosslinkable ends and micelle-reinforced SB hydrogels. (F) *In vivo* wound closure of the mechano-responsive hydrogel-based drug carriers [175]. (G) Structure and digital images of the CB-functionalized dextran hydrogel. (H) Wound healing efficiency of CB-functionalized dextran after 8 and 14 days [178].

The preparation of a hydrogel-based drug delivery system is complex because the synthesized polymeric products are expected to be nanosized to avoid physical obstructions in the human circulatory system (for instance, tight junction in the epithelial layer, mucus-carrier electrostatic interaction) and promote cell internalization. However, decreasing particle size is beneficial to promote a higher surface area, which results in more susceptibility to oxygen quenching and low efficiency of polymerization in nanogel fabrication. Accordingly, nanogel self-assembly through supramolecular interaction has been implemented to solve the problem. To control nanoparticular size and complex conformation, Singha and colleagues have prepared multiple-responsive hydrogels based on self-assembly via reversible addition-fragmentation chain transfer polymerization (RAFT) [168]. In the study, the CB polymer has been used as a macro-RAFT reagent and emulsion stabilizer to prepare a core-corona structure. As a result, the significance of corona-crosslinked motifs provides additional stability for gel structure owing to the multiple stages of covalent and physical interaction. With a more facile approach, Ye's group has utilized entirely physical SB hydrogel for local drug delivery (Fig. 10A and B) [169,170]. The charge screening effect resulted in chain dissociation and the degradation of the hydrophilic network due to the salt-dependent phase transition of the SB motif. Combined with fouling resistance, nanocomposite SB hydrogels exhibited excellent site-specific delivery through nanoscale tumor-targeting copper oxides and pH-triggered gel degradation [170]. As a result, the therapeutics effectively treated cancer mice, as evidenced by the reduction in tumor reoccurrence and tumor size as shown in Fig. 10C and D.

The mechanical performance of hydrogel-based drug delivery systems used as skin patches, internal wound sealants, and local delivery, such as the eyes, is crucial to ensure patient protection and comfort. To this end, a common strategy for enhanced mechanical properties is to increase crosslinker content during hydrogel curing process [171,172]. However, too little and overuse of the crosslinker content can diminish porous structure (low crosslinking density) or impede drug encapsulation and release capability (high crosslinking density). More importantly, hydrogels in both cases are susceptible to mechanical stress. To overcome the problem, additional physical bonds via catechol chemistry, nanocomposite, and micelle-mediated crosslinking have been recently exploited to induce self-healing capability and enhance mechanical performance, concurrently facilitate physical binding with drug molecule for sustained drug release [[172], [173], [174], [175], [176]]. For instance, nanoparticulate materials embedded in hydrogel-based contact lenses have also shown improvement in both mechanical performance and drug bioavailability of carriers. Hao and colleagues combined SB-based nanogel within hydrophilic network, showing a drug release profile for more than 10 days [174]. Zhang and colleagues demonstrated a novel therapeutic contact lens integrated with cationic heme-mimetic gallium porphyrin nanoparticles (Ga-CHP) [173]. Notably, contact lenses were reinforced with zwitterionic-elastomer hybrid, facilitating synergistic antibacterial strategies against bacterial keratitis: zwitterion motifs prevented bacterial fouling and contaminants from contacting injured eyes, while Ga-CHP exhibited bactericidal action via iron-blocking and photodynamic reactive oxygen species (ROS) production for 7 days. In acute wound treatment, Liu's group has introduced a mechano-triggered drug release system to ease pain (Fig. 10E and F) [175]. In the study, cross-linkable diarylated Pluronic F127 micelles were covalently bonded within SB-based network for drug encapsulation. Additionally, the micelles can also act as stress dissipating points, enabling the hydrogel to impressive tensile strain at 1420 %. Furthermore, upon compression or tension, these physical-crosslinking joints were temporarily ruptured, leading to drug release at wound site. This provides an excellent example that demonstrates the implementation of mechanical criteria in drug delivery applications.

Numerous diseases comprise multifactorial disorders, including oxidative stress, inflammation, and bacterial infections [177]. To this end, advances in zwitterionic biopolymers have shown their effectiveness toward comprehensive treatment. CB-functionalized dextran was further implemented in dermal wound healing for its antioxidation capability (Fig. 10G) [178]. Interestingly, CB conjugation on dextran provides an effective non-sticky interface to open wounds and enhances radical scavenging and potential anti-inflammatory capability for the gel owing to additional methylene protons in quaternary amine, accelerating wound healing process within 14 days (Fig. 10H) [178]. In a different approach, acrylate dextran was conjugated with SB monomers in situ via “thiol-ene” click chemistry toward enhanced fouling resistance of injectable hydrogels in cancer therapy [179]. From a future perspective, this implementation can be further extended to other polysaccharides, notably chitosan, and hyaluronic acid, for inclusive drug delivery systems for various diseases.

### 4.5 Other applications

Zwitterionic hydrogels have also been recognized in diagnostic applications. Owing to the non-specific fouling resistance, zwitterionic hydrogel coating can enhance the molecular sensitivity of decorated gold surfaces [180,181]. Luo and colleagues developed an electrochemical biosensor platform for the detection of prostate-specific antigen (PSA) in complex serum. In the study, physical deposition of amino acid-derived zwitterions was performed on an electrochemical biosensor based on poly(3,4-ethylenedioxythiophene) (PEDOT) to prevent non-specific protein fouling. Then, bioconjugation of anti-PSA on zwitterionic peptide hydrogel was executed via EDC/NHS reaction. As a result, the sensor system-based electrochemical signal depicted a linear response range from 0.1 ng mL^−1^ to 100 ng mL^−1^ with a signal-to-noise ratio of 3 and limit of detection 5.4 pg mL^−1 181^. For quartz crystal microbalance (QCM) measurement, Dai and colleagues have implemented surface-initiated atom transfer radical polymerization (ATRP) to form linear interpenetrating SB polymer into the rigid phenylboronic acid-grafted acrylamide network [180]. Harnessing mechanical toughness and durability of double network systems, the hydrogel coating demonstrated the feasibility of tough hydrogel for the protection and recycling uses of QCM sensors. Furthermore, interpenetrating network conformation was covalently immobilized on sensor chips, impeding the swelling behavior of the SB hydrogel under saline condition, stabilizing the continuous recording signal of QCM. Additionally, incorporating phenylboronic acid monomers provides a specific binding site for glucose. Combined with the strong ionic solvation of SB, this enables the detection of glucose in saliva samples, which is typically 27–1100 folds smaller than the glucose level in serum of diabetic patients.

The implementation of zwitterionic hydrogels can be realized in the preservation of circulating tumor cells (CTCs) at tumor sites and whole blood specimens. CTC represented a hallmark in non-invasive diagnostics and cancer progression monitoring, providing valuable information for assessment for long-course cancer treatment. Nevertheless, CTC diagnostic facilities are not widely available. To this end, Zhang and colleagues have recently developed extracellular matrix (ECM)-mimicking microgel platforms to protect a minor number of CTC from ROS overproduction of red blood cells and platelet activation [182]. In the study, two sets of CB microgels copolymerized with polyphenylboronic acid (BA) (set 1) and polyvinyl alcohol (PVA) were interblended with blood samples to form cell niche for CTC survival. Furthermore, the dynamic boronic ester bonds interconnecting boronic acid residues and hydroxyl groups improve the hydrophilic structural integrity of the 3D artificial CTC niche, provide ROS quenching capability, and inhibit platelet activation from blood deterioration. Furthermore, when comparing the microgels with marketed protectant reagents, the developed materials could maintain gene expression of CTC compared to normal cells.

## 5 Current challenges and future perspectives

Throughout the review, various synthesis techniques are examined from a material engineering perspective and summarized in Table 1. It is important to note that while these techniques have their advantages and disadvantages, careful design and appropriate application can minimize their limitations and maximize their potential in specific fields. In addition, while supramolecular-assembly hydrogels offer an effective strategy to avoid oxygen inhibition, preparing linear polymers with coupling agents or physical crosslinks necessitates complex chemical synthesis and a tedious process, limiting their industrial recognition. Nevertheless, this method is well-suited for research institutions and clinical facilities, offering controllable results for drug delivery, cell encapsulation, and tissue engineering. Although nanocomposite hydrogels are mechanically soft, they offer high flexibility and stretch, making them highly promising for wearable devices. The fabrication techniques for nanocomposite hydrogels have shown potential for scale-up manufacturing, as they are based on conventional single-network hydrogels and one-pot procedures.

Looking forward, there are still challenges to be addressed in the clinical translation of zwitterionic hydrogels, including batch-to-batch variation, long-term biosafety, and environmental concerns during the synthesis process. Among these issues, reproducibility is the most critical, particularly as bulk material engineering often requires specialized instruments like 3D and 4D printers. These challenges are even more pronounced for nano- and micro-scale hydrogels, leading to unpredictable results in drug delivery systems. Additionally, the high-water content of hydrogels (>90 wt%) can limit their application in extreme temperature conditions. In terms of the anti-polyelectrolyte effect, the mechanical properties of some zwitterionic hydrogels are sensitive to ionic strength, potentially compromising their anti-fouling and lubricity performance. In addition, given that ionic strength variations in complex electrolyte environments substantially impact mechanical stability, the selection of appropriate zwitterionic motifs becomes more critical than the choice of synthesis methodology when balancing mechanical robustness and biocompatibility in the development of zwitterionic biomaterial. Particularly, while SBMA motif excels at creating both affordable and mechanically tough hydrogels due to their distinctive phase transition behavior, they regularly experience reduced structural integrity in biological fluids or saltwater conditions. Fortunately, several strategies have been proposed to enhance salt resistance and anti-freezing properties in zwitterionic hydrogels, ranging from molecular engineering of novel TMAO motifs with zero carbon spacers to the use of additives such as glycerol-water binary solvent mixtures.

Another challenge in developing zwitterionic biomaterials is to achieve the correct balance of resistance to non-specific protein adsorption and promotion of specific biomolecular binding toward complete biomaterial integration to biological tissues. The problem is highly restrictive when the biomaterials are to be performed in complex media containing various serum proteins. The selective attachment of certain serum proteins, including albumin, fibrinogen and fibronectin, while deliberately preventing the non-specific adhesion of other proteins holds the key to unlocking the potential of bioactive anti-fouling materials [183]. Considering that carboxyl betaine motifs and other carboxylated zwitterions can undergo biofunctionalization, zwitterionic hydrogels decorated with ECM proteins (e.g., laminin, fibronectin and vitronectin) may promote cellular focal adhesion through integrin mediation, subsequently leading to cell spreading and growth [184,185]. Blending zwitterionic materials with polysaccharides has also shown significant progress in enhancing therapeutic benefits (e.g., anti-oxidation, anti-inflammation, antibacterial and anti-apoptosis), while enabling them with large-scale production for broader biomedical applications. Further bioactive properties of zwitterionic hydrogels can be supplemented with a wide range of dynamic covalent bonds (including catechol chemistry, click reactions, and boronic esterification), which have shown significant impact in cell encapsulation and growth. However, the balance between mechanical properties and functionality of dynamic covalent hydrogels in biological settings, as well as the insights of these biomaterials in response to physiological conditions, still requires long-term investigation.

## 6 Conclusions

In summary, the review addresses the challenges associated with the synthesis and fabrication of zwitterionic hydrogels for biomedical applications. The early sections discuss the selection criteria for zwitterionic motifs, emphasizing their importance in hydrogel formation. Following this, we explore the primary challenges in material manufacturing, mechanical fragility, and bio-inertness, which are also the focal points in the synthesis, fabrication, and functionality of hydrogels for biomedical use. To address these challenges, we present and discuss state-of-the-art research works on zwitterionic hydrogels. Overall, this review provides fundamental insights into the current challenges facing zwitterionic hydrogels, offering updated perspectives. We aim to encourage comprehensive considerations for material engineering that extend beyond lab-scale concepts and are applicable to scale-up production in industrial practices.

## CRediT authorship contribution statement

**Hoang Linh Bui:** Writing – original draft, Conceptualization. **Hoang Nam Nguyen:** Writing – original draft. **Jui-Yang Lai:** Writing – review & editing, Supervision, Project administration, Funding acquisition. **Chun-Jen Huang:** Writing – review & editing, Supervision, Project administration, Conceptualization.

## Ethics approval and consent to participate

Not applicable.

## Consent for publication

Not applicable.

## Funding

The authors acknowledge the 10.13039/100020595National Science and Technology Council (NSTC 113-2811-E−008 -009 -, 113-2918-I-008 -004-, 112-2221-E−008 -007 -MY3, 111-2628-E−008 -003 -MY3 and 111-2923-E−008 -004 -MY3) and 10.13039/501100002836Chang Gung University (OMRPD2N0011 and UERPD2Q0071) for financial support of this project.

## Declaration of competing interest

The authors declare that they have no known competing financial interests or personal relationships that could have appeared to influence the work reported in this paper.

## Data Availability

No data was used for the research described in the article.
